# Aquaporin-4 inhibition alters cerebral glucose dynamics predominantly in obese animals: an MRI study

**DOI:** 10.1038/s41598-025-99641-1

**Published:** 2025-05-05

**Authors:** Pablo Tirado-García, Adriana Ferreiro, Raquel González-Alday, Nuria Arias-Ramos, Blanca Lizarbe, Pilar López-Larrubia

**Affiliations:** 1 Institute for Biomedical Research Sols-Morreale (IIBM), Spanish National Research Council-Universidad Autónoma de Madrid, c/ Arturo Duperier 4, 28029 Madrid, Spain; 2 Department of Biochemistry, Universidad Autónoma de Madrid, Madrid, Spain; 3 Biomedical Research Networking Centre on Rare Diseases (CIBERER), Institute of Health Carlos III, Madrid, Spain

**Keywords:** Aquaporin-4, Mouse, Blood flow, Glucose, Obesity, Swelling, TGN-020, Magnetic resonance imaging, DTI, T_2_*, Biophysics, Neuroscience, Imaging, Computational biology and bioinformatics, Data processing, Biochemistry, Neurochemistry

## Abstract

Glucose uptake and metabolism are linked to microvascular blood flow and cellular swelling events, which are altered during obesity and can be quantified using magnetic resonance imaging (MRI). Aquaporin-4 (AQP4), the most abundant water-transporting transmembrane protein in the central nervous system, facilitates glucose transport and metabolism-derived water influx. However, its significance and regulatory capacity remain largely unknown. To better understand these processes, we acquired sequential diffusion tensor and T2*-weighted images of the brains of obese and non-obese mice, both before administering an AQP4 inhibitor and after a subsequent glucose challenge. We then subjected the resulting variables to principal component and linear mixed model analyses to assess the influence of diet, sex, administration of the inhibitor, and brain region on the data. Our findings indicate that AQP4-inhibited mice exhibit MRI values consistent with reduced microvascular blood flow and region-specific inhibition of glucose-induced cell swelling during obesity, highlighting a key role for AQP4 in glucose uptake and metabolism. Additionally, we observed that, prior to any experimental manipulation, obese mice displayed MRI signs of lower hippocampal blood flow and cerebral cellular anisotropy compared to controls, in agreement with vascular alterations and reactive gliosis processes.

## Introduction

**Fig. 1:**
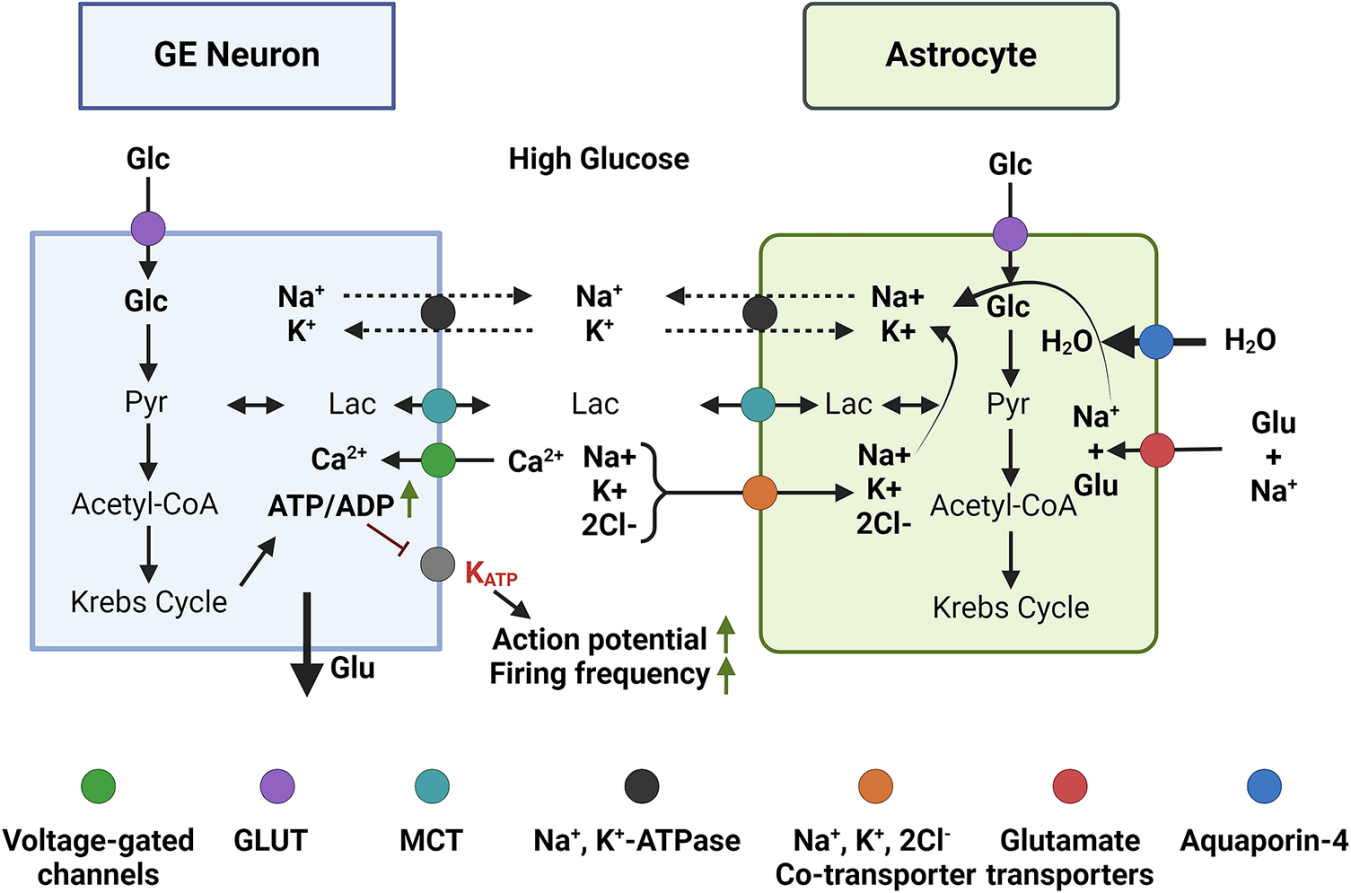
Ion flow between glucose-sensing neurons, astrocytes and the extracellular space. Glucose (Glc) enters neurons and astrocytes by GLUT transporters (purple) and is converted into pyruvate (Pyr). Pyr can be either converted into lactate (Lac) or enter the TCA cycle (Krebs cycle). Lactate is released by astrocytes and taken up by neurons through MCT transporters (cyan), where is reconverted into Pyr and can be used as a neuronal substrate for ATP and neurotransmitter production. In glucose-excited (GE) neurons (left panel, blue), high extracellular glucose levels and cellular uptake, changes ATP-ADP ratio, which causes the closure of the K_ATP_ channels, resulting in plasma membrane depolarization and Ca^2+^entry through voltage-gated channels (green), thereby increasing neuronal activity and neurotransmitter release. The released glutamate enters astrocytes with Na^+^ (red). To re-establish the concentration, Na^+^ leaves astrocytes via the Na^+^/K^+^-ATPase, with counter-transport of K^+^ or via the Na^+^/K^+/^2Cl^−^ co-transporter (orange). The entry of ions into astrocytes induces water to move intracellularly by AQP4 (blue).

Glucose is the main source of energy in the mammalian brain, and is critically important for ATP generation and neurotransmitter synthesis^1^. Its transport and metabolism are the result of a tight balance between microvessels, astrocytes, and neurons, the main components of the neurovascular unit^2^. Obesity, one of the most prevalent diseases of our era, is known to alter such elements and its corresponding equilibrium, including changes in the cerebral vasculature wall, in cerebral blood flow and glucose metabolism^3,4^, increased blood brain-barrier permeability^5^, and the development of astrogliosis and microgliosis^6,7^. Glucose entrance into astrocytes and neurons by their specific transporter channels, as well as the glucose metabolism-associated ion trafficking, involve water co-transport^8^, with water entering the intracellular space mainly by aquaporin-4 (**AQP4**), the most abundant water channel in the brain^9^. AQP4 is located in astrocytes and ependymal cells, is a key contributor of edema formation and resolution, and it is involved in astrocyte migration and astrogliosis^10^. Alterations in the expression and/or localization of AQP4 lead to a water homeostasis imbalance and have been associated with pathological conditions, including inflammation, stroke or traumatic brain injury^11^. Interestingly, during obesity, AQP4 expression has been reported to be augmented in some brain regions, which has been related to cerebral edema development^12,13^, but decreased in others, potentially due to particular ependymal cells-AQP4 downregulation after a worse glymphatic clearance^14^. During glucose transport and metabolism, water transport through AQP4 is known to yield a certain degree of astrocyte **swelling**^15–17^ (Fig. 1), but the extent of the regulatory capacity of such mechanism by AQP4, its influence on glucose metabolism and the corresponding alterations during obesity, remain to be understood.

Changes in extracellular cerebral glucose concentration are perceived by specialized glucose-sensing neurons, the glucose-excited (GE) or glucose-inhibited (GI), which alter their firing rate after a glucose stimulus. They have been described in the hypothalamus^18^ and in several brain nuclei that are part of the reward system, such as the hippocampus, thalamus and prefrontal cortex. Particularly, on GE neurons, high glucose uptake and the consequent augmented metabolism leads to an increase of the ATP/ADP ratio and the closure of the K_ATP_ channels, increasing neuronal activity, neurotransmitter secretion, and Ca^2+^ neuronal influx^19^ (Fig. 1). This augmented influx into the neurons and its elevated concentration on astrocytes, generates a dilation of the blood vessels^20^. Ionic trafficking in GE neurons may cause, again, astrocyte swelling via increased water entrance through AQP4 transport. Remarkably, glucose-sensing mechanisms are also perturbed during obesity^21^.

Magnetic resonance imaging (MRI) is a non-invasive imaging technique that provides anatomical and parametric information of structure, function and metabolism^22^. It is broadly used in the clinic as a diagnostic and prognostic tool of numerous brain pathologies such as ischemia, cancer or neurodegeneration^23^. The technique takes advantage of the magnetic properties of the water molecules’ hydrogen atoms, and particularly, diffusion weighted MRI (dMRI) uses the natural Brownian motion of water molecules to obtain information on the anatomical and biological structure of tissues^24^. Depending on the characteristics of such structures, the movement of water molecules can be isotropic, with no preferred direction, or anisotropic, indicating faster diffusion directions, such as in neuronal bundles. Diffusion tensor images (DTI) can measure this anisotropic behavior by quantifying parameters like mean diffusivity (MD, mean diffusion of the water molecules); axial diffusivity (AD, the diffusion along the fastest direction ); radial diffusivity (RD, the diffusion perpendicular to the main direction), and fractional anisotropy (FA, providing information about the extension of the anisotropy of water motion)^25^. Changes in water diffusion have been correlated with changes in AQP4 expression in central nervous system diseases, such as in the early stages of brain edema after hypoxic-ischemic/reperfusion injury, or obesity, which is known to show altered cerebral DTI parameters related to edema formation and neuroinflammation^26^. Diffusion MRI can also be used to tackle cerebral activation by revealing changes in the diffusion properties of water molecules independently to neurovascular coupling. Notably, glucose-induced swelling mechanisms have been reported by dMRI methods in the context of exogenous glucose administration^27^ or orexigenic activation^28^. The neuromorphological changes derived from neuronal activity processes are thought to be the cause of the dMRI changes reported during brain activation^29^, but its exact origin, including the potential contribution of activity-derived cellular swelling, time characteristics or sensitivity to monitor normal brain activity, remain to be clarified^30^.

T_2_*-weighted imaging (T_2_*WI) is an MRI acquisition able to detect small disturbances in the uniformity of the magnetic field, allowing detection of certain small lesions, and can be used for diagnostic purposes^31^, and detecting blood flow changes^32^. Briefly, the T_2_* relaxation time reflects how quickly the magnetization of water molecules decays due to magnetic field inhomogeneities and magnetic susceptibility differences within tissues. Local changes in the blood oxygen levels are the basis of functional blood oxygen dependent (BOLD) MRI^33^. Indeed, during neuronal activation, T_2_* changes are induced by a change in the ratio deoxyhemoglobin/hemoglobin. Deoxyhemoglobin is a paramagnetic molecule with an iron core that has 4 unpaired electrons per Fe atom, and its presence increases the local magnetic field, and affects T_2_*^34^. Interestingly, previous studies demonstrated brain changes in T_2_*WI images acquired during glucose uptake compared to a control^27^, suggestive of increased microvascular blood flow induced by glucose administration.

**Fig. 2:**
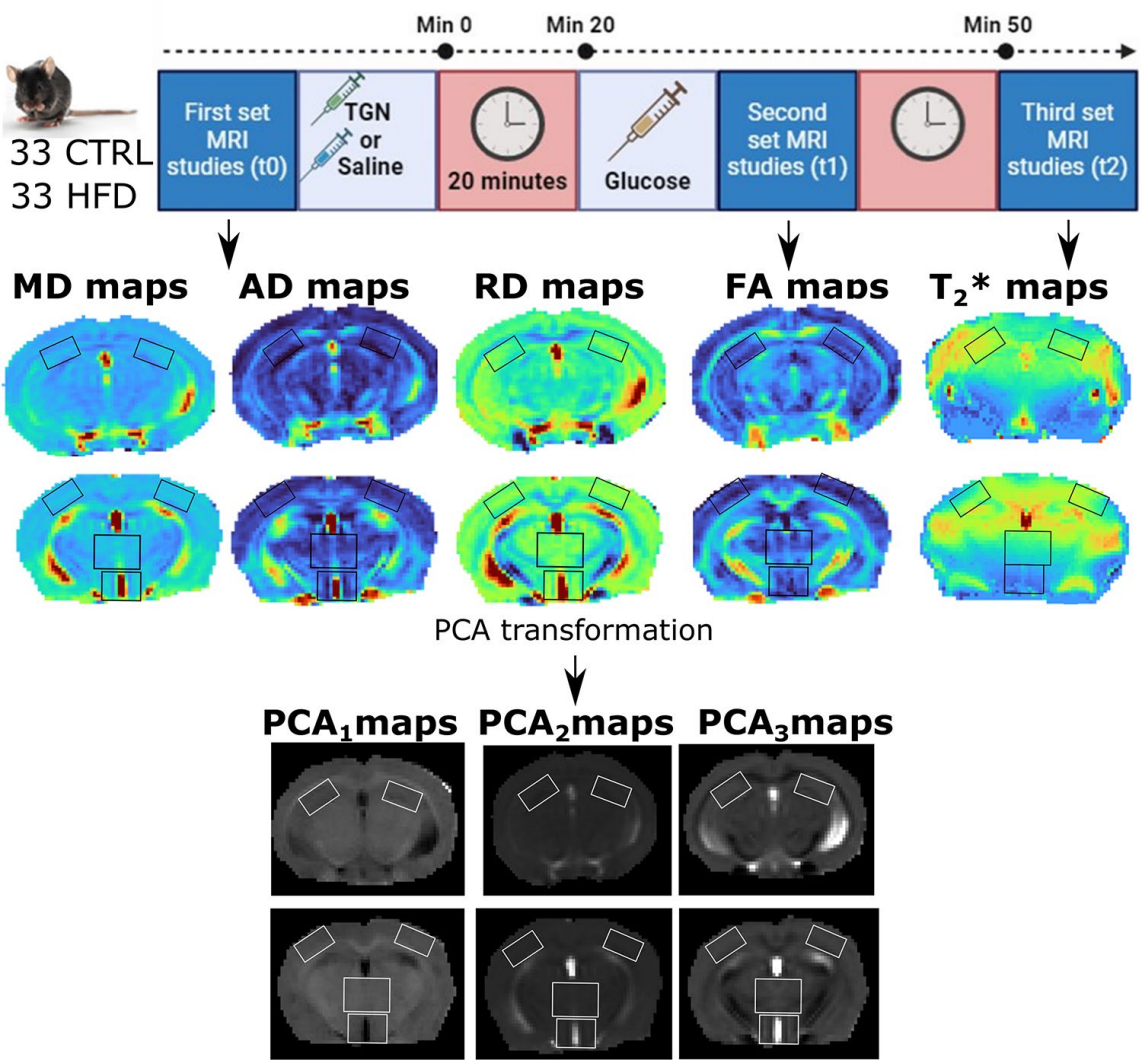
Experimental design and data analysis workflow. Upper panel: Temporal distribution of MRI studies and compound administration. The first set of MRI studies (t0) was acquired before any experimental manipulation. Subsequently, TGN (100 µL/25 g a 0.24 M, 0.96 mmol/kg administration) or saline (100 µL/25 g) were injected intraperitoneally (*i.p*.). After 20 min, glucose was administered (*i.p*., 200 µL/25 g, 2.08 M on a saline solution, 16.65 mmol/kg) and the second set of studies was performed (t1). 30 min later, the last set of acquisitions (t2) was acquired. Middle panel: Representative parametric maps of mean diffusivity (MD), axial diffusivity (AD), radial diffusivity (RD), fractional anisotropy (FA) and T_2_*. ROIs investigated are superimposed, including the left and right hippocampus, or right and left cortex, thalamus and hypothalamus. Lower panel: Example of representation of the PCA maps derived from the MRI parameters. After PCA transformation, each pixel and ROIs can be expressed in the new coordinates system.

On these grounds, in this study, we tested the hypothesis that the inhibition of the activity of AQP4 during a glucose challenge would reduce the concomitant swelling process and alter the dMRI effects. Moreover, we hypothesized that the effects on obese animals, where AQP4 expression, glial cells and glucose metabolism are perturbed, would be different. To test that, we acquired three consecutive DTI, and complementary T_2_*WI, to control or high fat diet (HFD) fed C57BL6 animals, before any experimental manipulation (“Basal”), twenty minutes after the administration of saline or 2-(nicotinamide)-1,3,4-thiadiazole (**TGN-020**), an AQP4 inhibitor which in animal models significantly reduces ischemic brain edema^35^, and immediately after a glucose insult (“Glc_short_”), or 30 min post-glucose (“Glc_long_”) (Fig. 2). We performed two complimentary data analysis of the cerebral MRI parameters. First, we assessed each MRI set separately (basal, Glc_short_ and Glc_long_), to unveil the specific effects of diet, sex or treatment (*HFD*, sex and TGN-20 effects on T2* and DTI at separated time points); next, we assessed how MRI the parameters evolved longitudinally in time for each animal, and considered the differences between the experimental groups (T2* and DTI follow up TGN-20 administration to HFD or CTRL mice). To do that, we applied principal component analysis (PCA) and subsequent linear mixed-model effects (lme) fitting to the predictor varianles: type of diet, sex, presence of inhibitor (“treatment”) and brain region. With this rationale, we sought to: (i) identify potential initial differences of DTI and T_2_*WI derived parameters between obese and non-obese mice, (ii) identify sex-related differences in the context of obesity, glucose uptake and AQP4 function, (iii) study the specific role of different cerebral regions during obesity, glucose uptake, and TGN-20 inhibition.

## Results

### Body weight

Male mice of the HFD group had significantly increased body weight (BW) than control animals, both in the “Saline + Glc” and “TGN + Glc” groups (45.1 ± 3.0 Vs 30.3 ± 1.7 for the saline batch, 49.0 ± 3.1 Vs 30.0 ± 1.4 for TGN) (p_adj_ < 0.005). Female HFD animals also depicted higher BW (32.1 ± 5.1 Vs 21.7 ± 0.3 for the saline batch, 30.6 ± 5.4 Vs 21.8 ± 0.4 for TGN) (p_adj_ < 0.05) (anova tests of BW Vs diet, sex and treatment with interaction), although BW differences where not that striking. The groups assigned to different treatment groups showed no significant differences between them.

### HFD, sex and TGN-20 effects on T2* and DTI at separated time points

**Fig. 3:**
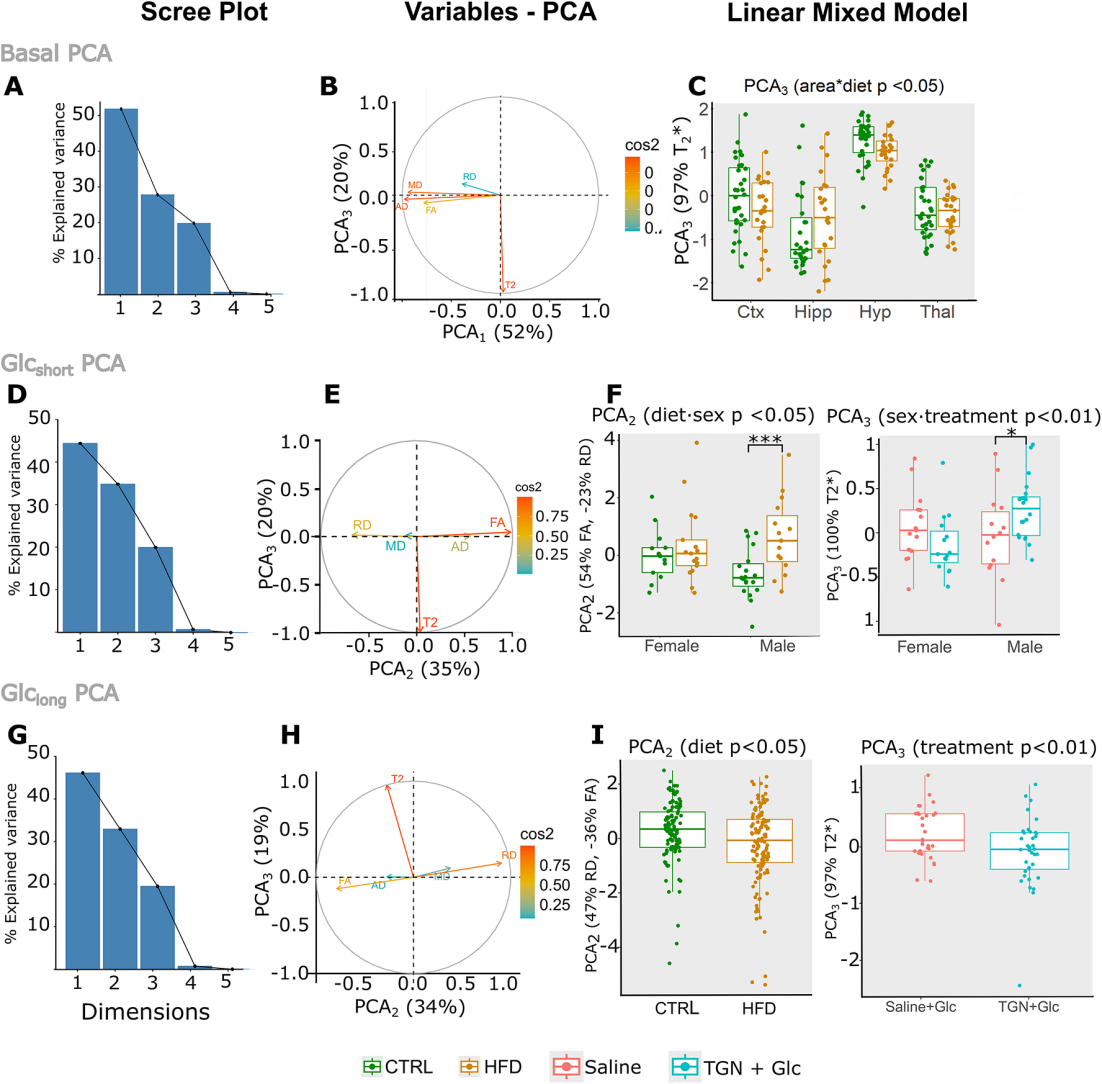
PCA and lme statistics. (**A**) Percentage of explained variances from each principal component (from PCA_1_ to PCA_5_), before TGN or saline administration (t0). Note how with 3 components, built as a linear combination of 5 MRI variables, the 100% of the variance is explained. (**B**) Biplots showing the contributions of the MRI variables to the first and third PCA dimensions (horizontal and vertical axis, respectively), as expressed by the squared cosines (cos2) of the coordinates. See how PCA_1_ has contributions from AD, MD, FA and RD (to a lesser extent) and that PCA_3_ is composed mainly by (−T_2_*), while (**C**) Results of linear mixed model statistics on the PCA_3_, and corresponding PCA_3_ mean values per animal and region on basal time, for control (green) or HFD (brown) mice, including animals form both sexes and treatment group. (**D**) Percentage of explained variances adding each dimension of the PCA at Glc_short_. Again, more than 90% of the variance is explained by only 3 variables. (**E**) Biplots of the contributions of the Glc_short_ MRI variables to PCA_2_ and PCA_3_ (horizontal and vertical axis, respectively), as expressed by the squared cosines (cos^2^ of the coordinates. Note how PCA_2_ has positive contributions from FA and AD, and negative additions from RD and MD, while PCA_3_ is still mostly T_2_*. (**F**) Values of PCA_2_ (left) and PCA_3_ (right), as a function of the predictor variables that have statistically significant effects. For PCA_2_, values for males and females fed with control (green) or HFD (brown) on time 1 were averaged between areas. Only the interaction *diet: sex* was significant (*p* < 0.05) and *post-hoc* tests showed increased PCA_2_ on males (p_adj_ < 0.001). PCA_3_ is shown as mean regional values for *vehicle + Glc* (red) or *TGN-020 + Glc* (blue) or at Glc_short_, for males and females. The significant interaction *sex: treatment* (*p* < 0.01) on PCA_3_ showed significant *post-hoc* tests increases with treatment only on males (*p* < 0.05) (right panel). (**G**) Percentage of explained variances adding each dimension, with three dimensions explaining again more than 90% of the variance. (**H**) Biplots showing the contributions of the MRI variables adquired at the last time point (Glc_long_), to the PCA_2_ and PCA_3_ components (horizontal and vertical axis, respectively), as expressed by the squared cosines (cos2) of the coordinates. In this case, RD contributes positively to PCA_2_, and FA and AD negatively, while PCA_3_ is mainly composed by T2*. (**I**) PCA_3_ mean values per animal at t_2_, for *vehicle + Glc* (red) or *TGN-020 + Glc* (blue), with a significant decrease in PCA_3_ on the *TGN-020 + Glc* animals (*p* < 0.01).

After performing one independent PCA procedure of the MRI variables per time point - the “Basal”, “Glc_short_” and “Glc_long_” PCAs -, we achieved a dimensionality reduction, with the first 3 PCA explaining ≥ 90% of the variance (Fig. 3A, D,G panels), for each time point, respectively. The component loads of the variables -the composition of the PCA, in terms of the original MRI-, was stable across the three time points, with AD and MD mainly composing PCA_1_, RD and FA PCA_2_, and T_2_* PCA_3_, (Table 1). Subsequently, the effects of the predictor variables -diet, sex or treatment- were tested on the three corresponding PCAs.

**Table 1:** Component loads of the variables at times 1, 2 and 3.

	PCA_1_ basal	PCA_2_ basal	PCA_3_ basal	PCA_1_ Glc_short_	PCA_2_ Glc_short_	PCA_3_ Glc_short_	PCA_1_ Glc_long_	PCA_2_ Glc_long_	PCA_3_ Glc_long_
MD	−0.58	0.29	0.03	−0.67	−0.08	0.01	0.61	0.29	0.10
AD	−0.61	−0.16	−0.04	−0.55	0.42	0.00	0.63	−0.21	0.00
RD	−0.24	0.77	0.12	−0.49	0.51	0.02	0.26	0.69	0.15
FA	−0.48	−0.52	−0.08	−0.10	0.74	0.04	0.39	−0.60	−0.12
T_2_^*^	0.02	0.15	−0.99	−0.02	0.03	−0.99	−0.06	−0.21	0.98

**Fig. 4:**
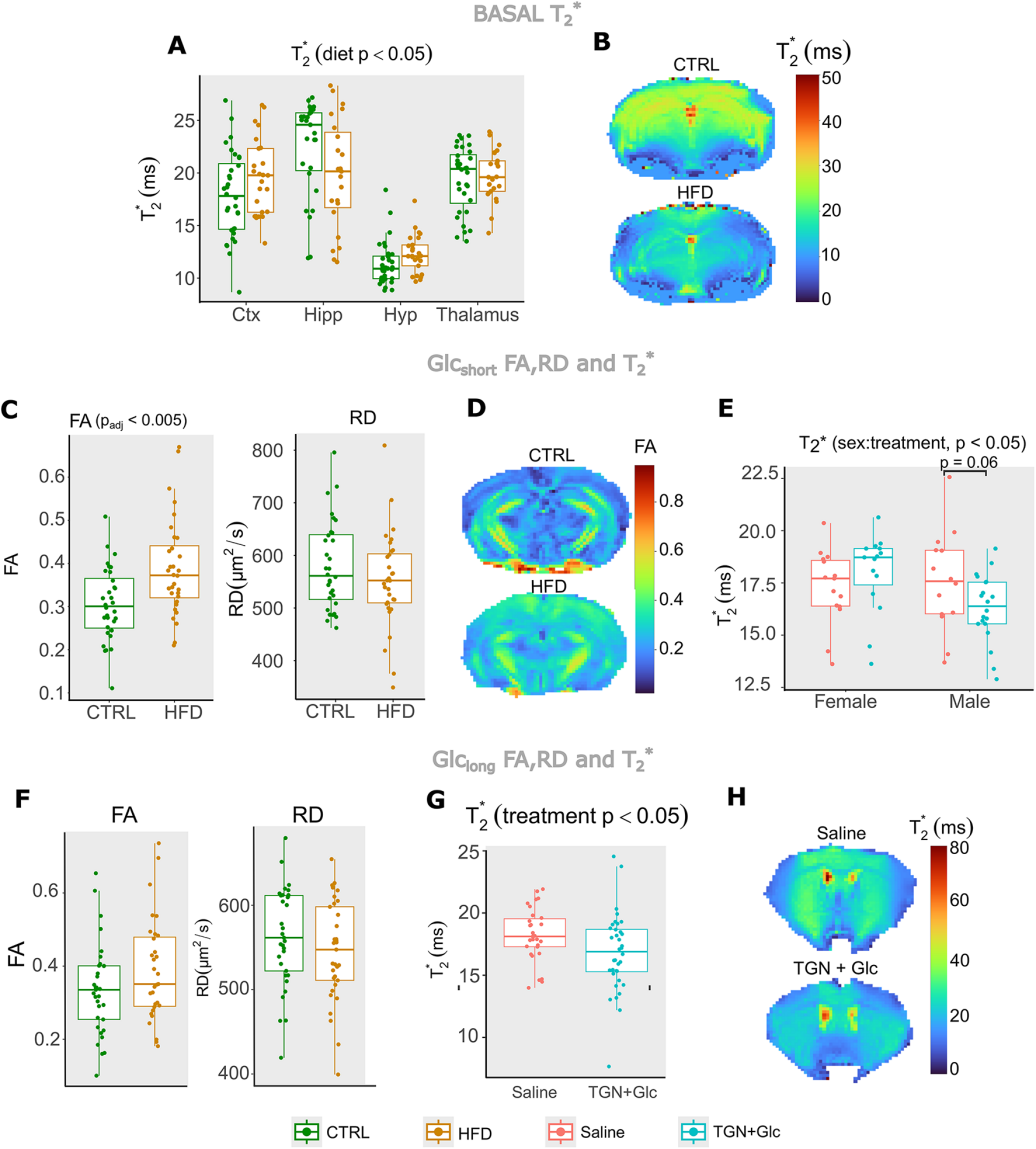
MRI changes with diet, treatment and sex: (**A**) T_2_* values for CTRL (green dots) and HFD (brown dots) mice, superimposed to a boxplot representation, for every region assessed. The effect of diet was significant on T_2_* (*p* < 0.05). (**B**) parametric maps of T_2_* values in the mouse brain, for a CTRL (top) and HFD (bottom) animals. Note the lower values of T2* in the HFD mouse. (**C**) FA and RD values for CTRL (green dots) and HFD (brown dots) mice (average values of four regions) during the Glc_short_ measurements, superimposed to a boxplot representation. The effect of diet was significant on FA (p_adj_ < 0.005). (**D**) Parametric maps of FA values in the mouse brain, for a representative CTRL (top) and a HFD (bottom) animal at the Glc_short_ time point. Note the higher FA values in the HFD mouse. (**E**) T_2_* values for saline + Glc (red dots) and TGN + Glc (blue dots) mice, superimposed to a boxplot representation, for males and females separately. The effect of the interaction sex: treatment was significant on T_2_* (*p* < 0.05). (**F**) FA and RD values for CTRL (green dots) and HFD (brown dots) mice (average values of four regions), superimposed to a boxplot representation, during the Glc_long_ measurements. (**G**) T_2_* values for saline + Glc (red dots) and TGN + Glc (blue dots) mice, superimposed to a boxplot representation, for Glc_long_. The effect of treatment was significant (*p* < 0.05). (**H**) Parametric maps of T_2_* values in the mouse brain, for a representative animal from the saline + glc group (top) and an animal from the TGN + glc batch HFD (bottom) animal, at the Glcs_long_ time point. See the lower FA values in the TGN mouse.

At basal time -before any administration- we found that the type of diet consumed affected significantly PCA_3_ (Fig. 3C), in a region-dependent manner (significant *region: diet*, *p* < 0.05, lme followed by Anova). Particularly, HFD mice showed lower PCA_3_ than CTRL mice, in all regions except in the hippocampus, with post-hoc corrected comparisons not reaching statistical relevance. PCA_3_ was composed mainly by T_2_* (Fig. 3B), and the specific analysis of **T**_2_* as a function of diet and region revealed similar results (*region: diet*, *p* = 0.05), with lower values on the hippocampus of HFD mice, and slightly higher in the rest of the regions, as compared to CTRL, and without reaching robust statistical evidences sub-regionally (Fig. 4A, B).

In the Glc_short_ measurements, the lme approach revealed a significant sex-dependent diet effect (*diet: sex*, *p* < 0.05) on PCA_2_ (Fig. 3F). Specifically, male HFD animals had higher PCA_2_, as compared to CTRL mice (Fig. 3F left). PCA_2_ was composed by FA and RD (Fig. 3E), and higher FA and lower RD could be seen in the HFD mice, respectively (Fig. 4C, D), with *p* < 0.05 only for FA (anova test of FA Vs diet, sex and *diet: sex*). Additionally, we found that the type of treatment affected PCA_3_, on a *region* and *diet*-dependent manner (interaction of *region: diet: treatment*, *p* < 0.05), and depending on sexes (treatment: sex, *p* < 0.05). Particularly, HFD animals with TGN treatment tended to higher PCA_3_, as compared to HFD saline-administered mice, in all regions except on the hippocampus, with adjusted post-hoc contrasts not reaching statistical significance. TGN-treated male animals showed significantly higher PCA_3_ values than the saline males (Fig. 3F right). Since PCA_3_ was mainly composed by T_2_*, this was translated into lower values of T_2_* in TGN-treated male animals, as compared to controls (Fig. 4E) (*p* < 0.05 *sex: treatment*, Anova tests, p_adj_ = 0.06 for post-hoc in males) .

In the Glc_long_ measurements (30 min after Glc administration) the type of diet was still affecting significantly PCA_2_ values (*p* < 0.05) (Fig. 3), with HFD mice having significantly lower PCA_2_ values than CTRL animals. RD and FA were positively and negatively correlated with PCA_2_, respectively (Fig. 3H), and lower PCA_2_ was translated into a lower RD and higher FA on HFD mice (Fig. 4F), but the independent tests of FA Vs diet, and RD Vs diet did not reach statistical significance. The type of treatment received had a significant effect on PCA_3_, (*p* < 0.01), with TGN + Glc administered mice presenting significantly lower PCA_3_ values, than Saline + Glc mice (Fig. 3I). Our results also revealed a significant *region*-dependent *diet* effect (region*diet interaction, *p* < 0.05) indicating that the HFD group had higher or equal PCA_3_ values than CTRL mice in all regions but the hippocampus (significantly only in cortex). Since T_2_* and PCA_3_ values were positively correlated (Fig. 3H), lower PCA_3_ with **TGN** indicated lower T_2_ (Fig. 4G, H) **(***p* < 0.05 t-test T_2_* Vs treatment**).**

### T2* and DTI follow up TGN-20 administration to HFD or CTRL mice

To quantify the animal-specific changes of the MRI parameters, before and after the administration of either “*saline + glc”* or “*TGN + glc”*, we built a global MFA that included MRI variables from the three longitudinal time-point measurements. Specifically, we included the subtraction, for each MRI parameter, of the “*Glc*_*short*_ *minus Basal”* values (Glc_short_-Basal), “*Glc*_*long*_ *minus Glc*_*short*_” (Glc_long_- Glc_short_) and “*Glc*_*long*_ *minus Basal*” (Glc_long_- Basal) and performed the MFA analysis on them. Results yielded 4 relevant principal components, and, subsequently, the effects of *diet*, *region*, *sex* or *treatment* were assessed by a lme approach followed by Anova. After applying such procedure, we observed a common regional pattern, with the hippocampus showing consistently a different tendency, as compared to rest of the regions (data not shown). We thus decided to consider the hippocampus as an independent region and conducted a specific MFA for the hippocampus, and a MFA from the rest of the regions.

### Hippocampal evolution

The MFA on the hippocampus resulted in 4 PCAs explaining ≥ 80% of the variance, thus achieving a dimensionality reduction from 12 initial MRI parameters to 4 main components..PCA_1_ (30% explained variance) was mostly composed of variables from the time regimes Glc_long_-Basal and Glc_short_-Basal, and a PCA_2_ (20%) mainly composed by the time regime Glc_long_-Glc_short_. PCA_3_ contained a mixture of three-time regimes, and PCA_4_ was formed by Glc_short_-Basal and Glc_long_-Basal. The component loads of the experimental MRI variables on the global PCA, are summarized in (Table 2).

**Table 2:** Hippocampal MFA component loads of the experimental variables.

	PCA_1_	PCA_2_	PCA_3_	PCA_4_
MD_Glcshort−B_	0.67	−0.32	0.52	0.39
AD_Glcshort−B_	0.80	−0.40	0.36	−0.10
RD_Glcshort−B_	0.09	−0.01	0.45	0.88
FA_Glcshort−B_	0.70	−0.39	0.07	−0.54
T_2_^*^_Glcshort−B_	−0.14	0.34	0.26	−0.19
MD_Glclong−B_	0.89	0.37	0.10	0.23
AD_Glclong−B_	0.77	0.39	0.46	−0.13
RD_Glclong−B_	0.71	0.22	−0.33	0.56
FA_Glclong−B_	0.29	0.14	0.75	−0.55
T_2_^*^_Glclong−B_	−0.43	0.34	0.12	0.01
MD_Glclong−Glcshort_	0.44	0.79	−0.41	−0.10
AD_Glclong−Glcshort_	−0.03	0.96	0.12	−0.03
RD_Glclong−Glcshort_	0.65	0.23	−0.69	−0.12
FA_Glclong−Glcshort_	−0.43	0.54	0.67	0.01
T_2_^*^_Glclong−Glcshort_	-0.43	0.14	-0.08	0.17

On PCA_1,_ the most contributing variables were (MD 16%, AD 12%, RD 10%) _Glclong−B_, (AD 13%, FA 10%, MD 8%)_Glcshort−B_ and (RD 10.5%, FA 10%, MD 8%)_Glclong−Glcshort_, all with a positive contribution. The type of treatment received had a significant effect on PCA_1_ (*p* < 0.05), with the TGN + Glc group showing higher (and positive) PCA_1_ values, as compared to Saline + Glc animals, which had negative PCA_1_ values (data not shown). Since PCA_1_ was composed by changes of MD, AD, RD and FA, positively correlated, negative PCA_1_ in the Saline + Glc animals can be translated into decreasing MD, AD, RD and FA from basal to post times, and from basal to pre, but augmenting TGN + Glc group.

On PCA_2_, the most contributing variables were (AD 34%, MD 22.5% and FA 10,5%), all from the Glc_long_-Glc_short_ regime. A significant *diet*-dependent treatment effect (interaction of *diet: treatment*, *p* < 0.001) was revealed. On HFD mice, TGN treatment group showed significantly lower (and negative) PCA_2_ values than saline animals, which had positive PCA_2_ values (Fig. 4A). Since PCA_2_ represented changes of AD, MD and FA, positively correlated, a positive **PCA**_**2**_ in the Saline + Glc mice can be translated into augmenting AD, MD and FA values in the time regime from Glc_short_ to Glc_long_ (Glc_long_ – Glc_short_ > 0) but decreasing (Glc_long_ – Glc_short_ < 0) on the TGN + Glc group (Fig. 4B) (diet: treatment p_adj_ < 0.05 for AD and FA, and subsequent post-hoc padj < 0.05 for treatment effects only on HFD mice).

No relevant effects were found on the effects of diet or treatment on PCA_3_ and PCA_4_ on the hippocampus.

### Cortex, thalamus and hypothalamus evolution

MFA on the hippocampus resulted in 4 PCA explaining the target variance with a PCA_1_ (31% explained variance) formed by Glc_long_-Basal and Glc_short_-Basal, a PCA_2_ (23%) mainly composed of variables from the time regime Glc_long_-Glc_short_ and Glc_short_-Basal. PCA_3_ (18%) was mostly composed of the time regimes Glc_long_-Basal and Glc_short_-Basal and PCA_4_ (14%) formed by Glc_long_-Glc_short_ and Glc_long_-Basal. The specific component loads of the MRI variables on the *rest of the regions* are summarized in (Table 3).

**Table 3:** *Rest of the regions* MFA component loads of the experimental variables.

	PCA_1_	PCA_2_	PCA_3_	PCA_4_
MD_Glcshort−B_	0.29	−0.74	0.52	0.24
AD_Glcshort−B_	0.73	−0.66	−0.00	0.00
RD_Glcshort−B_	−0.41	−0.31	0.75	0.34
FA_Glcshort−B_	0.82	−0.35	−0.38	−0.19
T_2_^*^_Glcshort−B_	0.08	−0.27	0.17	0.16
MD_Glclong−B_	0.68	0.16	0.69	0.01
AD_Glclong−B_	0.92	0.13	0.14	0.32
RD_Glclong−B_	0.01	0.10	0.90	−0.34
FA_Glclong−B_	0.78	0.06	−0.35	0.47
T_2_^*^_Glclong−B_	0.00	−0.17	0.36	0.02
MD_Glclong−Glcshort_	0.10	0.90	0.18	−0.21
AD_Glclong−Glcshort_	0.17	0.90	0.15	0.35
RD_Glclong−Glcshort_	0.47	0.45	0.13	−0.73
FA_Glclong−Glcshort_	−0.14	0.54	0.07	0.81
T_2_^*^_Glclong−Glcshort_	−0.07	0.04	0.29	−0.12

On PCA_3_, the most contributing variables were RD and MD (Glc_long_-Basal 30% and 17.5%, respectively) and RD and MD (Glc_short_-Basal 20,5% and 10%). Our lme showed a significant *sex* and *region*-dependent *diet* effect (interaction of region*diet*sex, *p* < 0.05), where the HFD group tended to have lower or equal PCA_3_ values.

No relevant effects were found on the effects of diet or treatment on PCA_1_, PCA_2_ and PCA_4_ on the cortex, thalamus and hypothalamus.

## Discussion

In this work, we investigated the role of AQP4 during glucose uptake in obese and non-obese mice by MRI in four brain regions. Specifically, we performed three sequential DTI and T_2_*WI acquisitions to follow the effects of TGN (or its vehicle, saline) administration prior to the glucose insult. Next, having obtained a series of MRI parameters per region and time point, we applied PCA and MFA approaches to the data to reduce dimensionality, and then used linear mixed models on the corresponding main components to infer the potential effects of type of treatment, diet consumed, sex or brain regions, on the MRI variables.

### Effects of TGN on microvascular blood flow and swelling

**Fig. 5:**
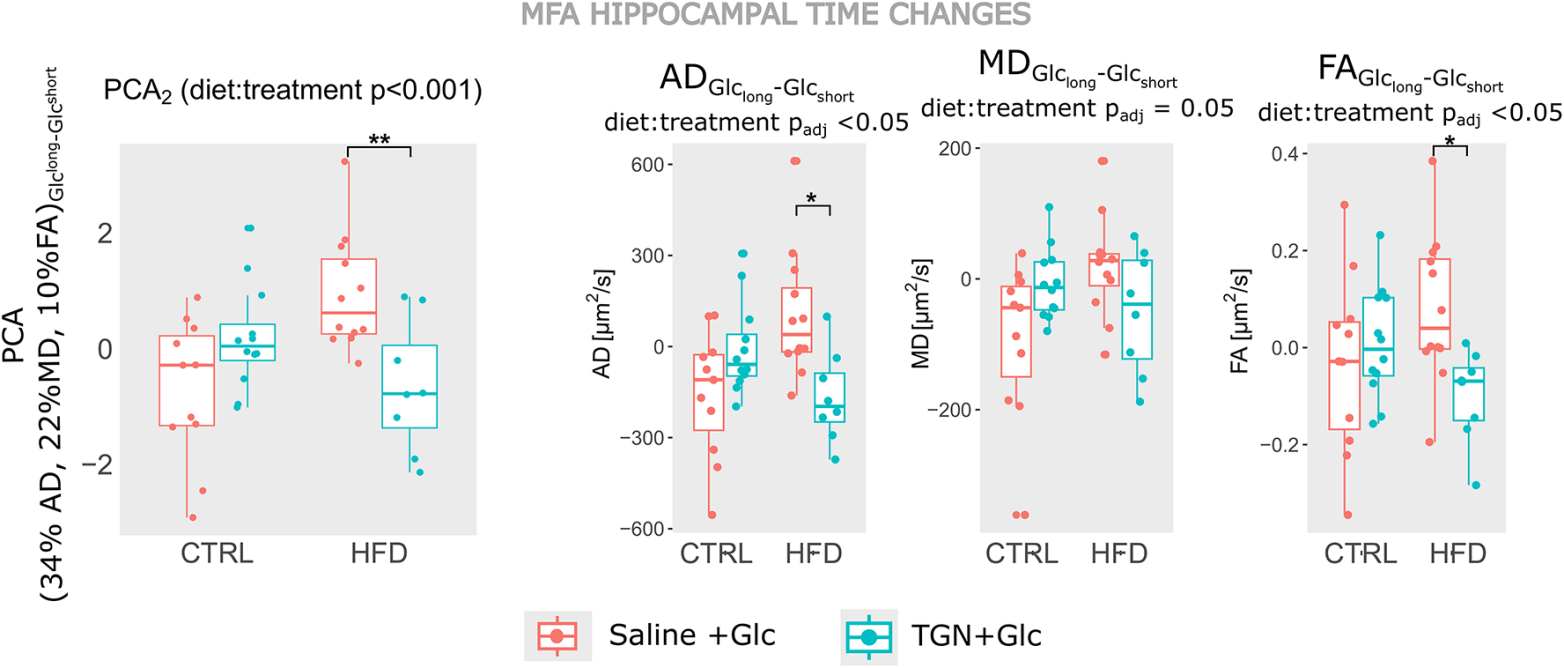
MFA Hippocampal time changes during a glucose insult with or without AQP4 inhibition. (**A**) Hippocampal PCA_2_ mean values of CTRL and HFD mice for the *vehicle + Glc* (red) or *TGN-020 + Glc* (blue) groups, as found with MFA, where the interaction *diet: treament* showed significant effects on PCA_2_. Post-hoc tests depicted relevant differences only on obese animals (*p* < 0.01). (**B**) Main MRI variables underlying hippocampal PCA_2_, depicting the experimental AD, MD and FA changes between Glc_short_ and Glc_long_, particularly values from the “long” subtracted by the “short”.

Our results are consistent with a change in the microvasculature blood flow induced by glucose that is inhibited by TGN, as quantified by lower T_2_* values in TGN mice, as compared to saline animals, (Figs. 3F and I and 4E, G and H). Such effects were more pronounced in males, during the Glc_short_ measures, but the sexual dependance disappears in the Glc_long_. In parallel, our data agrees well with a glucose-induced cell swelling mechanism in the hippocampus of HFD mice that is inhibited by TGN, as detected by higher FA, AD and MD changes from Glc_short_ to Glc_long_ in saline animals, as compared to TGN-treated mice (Fig. 5). Additionally, our work reports specific diet effects on brain cellularity, with HFD mice exhibiting lower RD and higher FA than controls, more remarkably in males (Figs. 3F and 4C), which is consistent with the presence of gliosis^36^. Notably, this diet effect on diffusivity was still remarkable after the long effect of glucose (Figs. 3I and 4F).

Considering both the T2* and DTI measures, results are consistent with a TGN reduction of the microvascular blood flow and cerebral activity that is detectable by MRI, highlighting the role of AQP4 during glucose uptake. Indeed, previous studies showed that glucose administration leads to increased cerebral activity and microvasculature blood flow^27^, and that such changes can be detected by MRI as increased markers of cellular swelling and microvascular blood flow, probably through glucose-sensing neurons^37^. Glucose uptake in the CNS is specifically associated with the entry of water into the cells via AQP4, leading to cell swelling and a decrease in extracellular space^15,38^. When glial swelling occurs, the cell shape becomes elongated^39^, resulting in more anisotropic diffusion of the water molecules, and MRI has been used to detect gliosis processes during obesity^40^. Particularly, using DTI, changes in cell shape can be characterized by higher FA^41^, higher AD and MD, and lower RD^42^. TGN-020 is known to inhibit AQP4 channels, preventing the passage of water through them^43^. Such inhibition may cause an alteration in the flow of ions (K^+^, Ca^2+^, etc.), altering astrocyte function and thus impairing the glucose-induced swelling. Our results revealing in vivo the relationship between AQP4 inhibition and impaired changes are consistent with previous studies, which reported ex vivo how the activity-dependent glial swelling was impaired on AQP4 knockout mice^44^, or in vitro how the deletion of AQP4 reduces astrocyte swelling during an oxygen-glucose deprivation challenge^45^.

### Diet effects

Our work reveals that diet affects the T_2_* and DTI values of the mouse brain. For instance, we report region-specific altered T_2_* values on obese mice at basal measurements, as compared to non-obese, suggesting that HFD induced lower values specifically in the hippocampus, on agreement with previous results in humans. Indeed, obesity is associated with both generalized and region-specific reductions in cerebral blood flow, with the hippocampus being a particularly vulnerable area. Previous neuroimaging studies demonstrated that higher body mass index correlated with progressively reduced blood flow across nearly all brain regions, indicating a global decline in perfusion^46^. The hippocampus—critical for memory and linked to Alzheimer’s disease (AD) risk shows pronounced sensitivity, with obesity-related hypoperfusion comparable to aging effects^47^.

The experiments here reported also highlight that the swelling-impairment effects of TGN depend on the diet consumed, as found in the results of the MFA, again in the hippocampus (Fig. 5). On the Glc_short_ to Glc_long_ period -the interval when glucose is expected to act on the brain- the TGN group had lower FA, MD and AD changes, as compared to the saline animals, only on the HFD subgroup. These results are consistent with the inhibition of a directionally dependent, glucose-induced swelling process that would occur in saline, and the fact that this change was reported only on the HFD group may indicate that AQP4 is more effectively inhibited in this group. Moreover, is consistent with previous experiments that have reported increased AQP4 expression during obesity^12,13^.

### Sexual dimorphism

In this study, we reported some sexual differences, including an increased susceptibility to diet, regarding the effects on brain DTI (Fig. 3F left), and to treatment changes on T_2_* (Fig. 3F right), in male mice. In animal models of diet-induced obesity, sexual differences are common, with males developing the obese phenotype typically faster and to a higher degree than females, who seem to be more protected against its development^48^. This is reflected, for example, in the BW of the animals, in which diet induces larger increases in the case of males, as reported here. Likewise, brain changes underlying the obese phenotype are sexual-dependent, with male mice being more prone to depict a neuroinflammatory profile^49^. In this sense, our results are consistent with an increased male-specific presence of astrogliosis and microgliosis during HFD. At the same time, our results suggest that TGN inhibition may be more effective under circumstances of increased AQP4 abundance.

#### PCA and MFA

In this work, the use of PCA and MFA allowed a reduction in dimensionality, obtaining new principal components that were independent from each other. This implies that the statistical tests performed on the corresponding components were independent^50^, and allowed us to pinpoint those combinations of MRI variables that were affected by predictor variables. Briefly, for example, from the four DTI-derived variables (MD, AD, RD and FA), which are highly correlated between them, the PCA method retained only two relevant components, with MD and AD showing the highest scores on PCA_1_, and RD and FA dominating PCA_2_. The statistical tests on PCAs revealed that only PCA_2_ resulted remarkably altered by the explanatory variables, particularly by diet and sex, and we could not find any treatment effects. This led us to explore in detail the significance of the variables sex and diet on FA and RD, and no further tests on MD or AD were performed, and the variable treatment was omitted (Fig. 4), thus reducing the number of tests performed and the probability of committing type I errors. Interestingly, in some cases, lower p-values were found on the tests on the created components (PCA_2_) than on the tests of the MRI variables composing them, suggesting the convenience of the method to find the variable combination that best reveals the underlying effects. In our study, PCA_2_ captured the variance related to smaller diffusivities (RD < AD), and to the degree of diffusion anisotropy, and thus to the degree on anisotropy of cells that shape such movements. The consistent negative correlation between FA and RD suggests that RD alterations may be good indicators of potential changes on cellular shape, in agreement with previous literature^36^. T_2_*, on the other hand, was in the three time-points almost exclusively dominating PCA_3_, showing lower correlations with the diffusion behavior of water molecules and potentially reflecting the vascular components.

MFA, on the other hand, gave us information on how the time periods, and the variables within, were correlated between them. By definition, MFA builds first separated PCA for each time regime, and after corresponding normalizations, generates a global PCA in which the initial variables are expressed as function of such global PCAs^51^ (Tables 2 and 3). In our results, both in the *MFA hippocampus*, and in *rest of the regions* analysis, PCA_1_ was mainly dominated by variables from the “Glc_short_*-*basal” and “Glc_long_-basal” regimes, with MD and AD showing the higher loadings. On the contrary, the MRI variables with the higher PCA_2_ loadings corresponded to the “Glc_long_-Glc_short”_ interval, with a clear domination of AD, followed by MD. Considering the experimental timeline of TGN/saline and glucose administrations followed here, PCA_1_ seems to be expressing potential differences on global diffusivity caused by the TGN administration itself, while PCA_2_ may be more related to the variance induced by glucose uptake and metabolism, and its effects on the highest diffusion directions.

## Limitations

In this work, we used TGN-020 as an inhibitor of AQP4 function, based on evidence of several previous studies^35,52–54^. However, some research has reported the inability of this compound to inhibit AQP4 function^55^, highlighting the need for a clearer understanding of the TGN-020 potential targets before interpreting the results of the experiments in vivo. In our study, based on MRI measurements, TGN administration appeared to effectively inhibit AQP4 channels, since it prevented the glucose administration-induced MRI changes observed in the absence of the inhibitor, the cellular swelling. While our results correlate well with previous studies reporting such swelling inhibition^44,45^ future experiments should investigate the mechanistic insights of the MRI-detected processes.

There are several limitations of the PCA or MFA approaches carried out with our data. In both cases, the underlying correlation of the initial MRI variables was not that strong (Kaiser-Meyer-Olkin criteria about 0.5), which effectively meant reducing only from 5 to 3 the number of components. On the MFA method, on the other hand, since some animals did not have information on the three time points, due to experimental problems, some data was inevitably lost.

Regarding the DTI variables, not all animals were acquired with the same number of directions, as specified in the methods section. Indeed, those animals acquired during the first batch included acquisitions of 6 directions only. This group showed lower data quality, which could add a potential bias in interpretation. It should be noted, however, that the type of study (“batch 1” or “batch 2”) was initially included as a predictor variable in the lme approaches, to test is belonging to either of the groups was affecting significantly the PCA variables, and no relevant effects were reported (data not shown).

While our findings offer valuable insights into AQP4 inhibition and glucose metabolism in the context of obesity, it is important to recognize the limitations of using a mouse model to extrapolate results to humans. One significant limitation is the difference in basal metabolic rates between mice and humans, which may affect generalizations related to energy expenditure and glucose regulation. Additionally, while the DIO models replicate key features of human obesity, including glucose intolerance and fat accumulation, the precise metabolic pathways and hormonal responses, particularly those related to glucose sensing and insulin signaling, may differ between the two species. Furthermore, the inflammatory response in mice is often more acute, and the distribution of fat (subcutaneous vs. visceral) may vary, influencing how obesity-related inflammation impacts glucose metabolism and brain function. Although the glucose loading model used in this study helps to mimic postprandial glycemic responses, its exact relevance to human physiology warrants careful consideration. In humans, glucose metabolism and uptake are regulated by a more complex set of hormonal controls.

Isoflurane anesthesia can significantly influence cerebral perfusion by inducing vasodilation and altering cerebral blood flow, as reported in previous studies. In our study, we maintained a consistent anesthetic protocol for all animals, ensuring that any observed differences between experimental groups were not confounded by variations in anesthesia depth. While isoflurane anesthesia may influence the absolute values of perfusion-related parameters, our analysis focused on relative differences between conditions (e.g., glucose administration and AQP4 inhibition), which remain interpretable within this controlled setting.

Although the findings should be interpreted within the context of an anesthetized model, the use of isoflurane was necessary to ensure stability and immobility during MRI acquisition, which is critical for obtaining high-quality, artifact-free images. While awake imaging could avoid the confounding effects of anesthesia, it introduces other challenges, such as motion artifacts and stress-related physiological changes, which could also confound the interpretation of MRI-derived metrics.Finally, another limitation is that while our study demonstrated the effects of TGN-020 in inhibiting AQP4 and altering brain microvascular blood flow in mice, these results may not directly translate to human neurovascular function. The expression and regulation of AQP4 in human brain tissues might differ, affecting the applicability of our results to human conditions.

## Conclusions

We can conclude that TGN-020 acts as an AQP4 inhibitor after a glucose insult that results in changes in the neurovascular unit detectable by MRI in vivo, including lower blood flow in several brain regions, as detected by T_2_*WI, and a decrease in glucose-induced cell swelling process detectable by DTI in the hippocampus of obese mice. In addition, we observed in obese mice MRI indicators of altered hippocampal microvasculature blood flow, potentially related to vascular inflammation, and DTI markers consistent with astro- or microgliosis processes, predominantly on males.

## Materials and methods

### Experimental design

The mice used in this study were bred and housed in our institutional animal facility at Institute for Biomedical Research Sols-Morreale (Reg. No. Es280790000288). *n* = 66 adult C57BL6/J mice (32 females) were kept in a room of the animal facility with controlled humidity (47%) and temperature (21–23 °C), a 12 h light/day cycle and fed with a standard laboratory food (SAFE DIETS). At 6 weeks of age, 33 animals (18 females) were switched to an HFD (60% fat, 20% carbohydrates, 20% proteins from D12492, Research Diets, New Brunswick, NJ), to induce obesity, and the rest continued with standard diet. After 18 weeks of diet diversification, mice were subjected to MRI under anesthesia provided through a nose mask (isoflurane 1-1.5% in O_2_, 1 L/min) during the whole study, to assess the response to a glucose administration stimulus (i.p., 200 µL/25 g, 2.08 M on a saline solution, 16.65 mmol/kg) preceded by a saline i.p. (100 µL/25 g) or TGN-020 (100 µL/25 g a 0.24 M, 0.96 mmol/kg administration), 20 min before the glucose insult^56^. Depending on the diet and the type of administration received, animals were thus assigned to four groups of analysis, (i) fed with a standard laboratory control diet and injected first with saline and glc (7 males and 7 females), (ii) fed with CTRL and administered with TGN-020 and glc (11 males and 7 females), (iii) group fed with HFD and administered with saline and glc (6 males and 7 females), and (iv) group fed with HFD and administered with TGN-020 and glc (8 males and 7 females) (Fig. 2). Once the MRI experiments finished, animals were euthanized by rapid decapitation, still under the effects of anesthesia administered during the imaging sessions.

### Compound preparation

TGN-020 is water-insoluble; therefore, a sodium salt derivative of the compound (TGN-Na) was prepared to facilitate its subsequent injection into animals using a saline solution. First, 41.20 mg of TGN-020 was placed in a round-bottom flask with 4 ml of Millipore water. Next, 426.24 µL of NaOH (1 M) were slowly added, leaving it stirring for 30 min at room temperature. Finally, the solution obtained was filtered and then lyophilized to generate the sodium salt (TGN-020-Na) that was injected into the animals (throughout the text, this sodium salt will be referred to as TGN-020).

### MRI acquisitions

Mice were subjected to MRI under anesthesia (isoflurane) using a superconducting magnet with 16 cm diameter and a gradient insert of 360 mT/m (Biospec^®^ 7T, Bruker Biospect, Ettlingen, Germany), in two separated animal cohorts. First, DTI studies (6 directions, b-values = 400 µm^2^/s & 1800 µm^2^/s, field of view = 23 × 23 mm^2^, slice thickness = 1.25 mm, 5 slices) and T_2_*WI to generate T_2_* maps (TR = 300 ms, flip angle = 30, 10 echoes, first TE = 2 ms, inter echo time: 4 ms, 8 averages, and the same geometrical parameters as DTI) were acquired in an initial group of animals (*n* = 35, *n* = 19 with DIO, *n* = 15 females). Subsequently, an additional cohort was investigated (*n* = 31, *n* = 14 with DIO, *n* = 17 females) with DTI (15 directions, b- values = 400 µm^2^/s & 1800 µm^2^/s, field of view = 23 mm, slice thickness = 1 mm, 5 slices) and T2*WI with the same conditions of the first batch. In both cases, brain slices were positioned to contain the hypothalamus, thalamus and cortex on the center acquisition slice, while the frontal hippocampus remained located in a contiguous slice. Regions were identified with the help of anatomical atlas^57^ For each mouse, three DTI and T_2_*WI blocks were acquired, at basal (t = t0), 20 min after TGN (or saline) administration and immediately after the glucose insult (t = t1), and 30 min after the glucose administration (t = t2) (Fig. 2).

### Image processing

DTI and T_2_*WI of each mouse were processed using Resomapper, a home-made software based on Dipy^58^. Pre-processing algorithms included a noise reduction filter (Patch2self) for DTI and the *adaptive soft coefficient matching* filter for T_2_*WI, both used to improve image quality and selected based on previous literature^59^. Processing of the DTI images lead to the obtention of MD, AD, RD and FA parametric maps, and by processing the T_2_*WI, T_2_* maps were obtained. Next, ImageJ software (U. S. National Institutes of Health, Bethesda, Maryland, USA, https://imagej.nih.gov/ij/) was used to manually select the regions of interest (ROIs), including: the hypothalamus (120 voxels), thalamus (140 voxels), cortex (144 voxels) and the hippocampus (110 voxels)(Fig. 2). ROI selection was performed blindly, and corresponding pixel coordinates of each subregion were saved and stored as .*txt* files.

### Data filtering and preparation

**Matlab** software (R2010b, MathWorks Inc., Natick, MA) was used to overlay all ROIs on all images of each mouse. Briefly, by automatically reading the coordinates from the Image-J derived .*txt* files, the information was inferred to use the same coordinates on all diffusion and T_2_* maps. Datasheet documents were then generated to include the information on MD, AD, RD, FA and T_2_* values for each pixel. The information regarding the corresponding subregion, time-point of acquisition (t0, t1 or t2), animal identification, type of diet, sex and type of treatment of each pixel was added in separate columns, resulting in a unique datasheet containing all the MRI information at a pixel level.

Using home-made R-scripts, data filtering and restructuring were performed. From the pixel datasheet of MRI generated by Matlab, pixel values were filtered to exclude the potential contamination of the CSF from ventricles, by removing those pixels with MD, AD and RD values > 1300 mm^2^/s or T_2_* values > 30 ms, as previously described^49^. Additionally, pixel outliers from each animal subregion (mean +/− 1.5 - interquartile range) were eliminated.

### Principal component analysis (PCA)

Aiming for dimensionality reduction, MRI variables were transformed via PCA (Fig. 2). PCA is a mathematical algorithm that reduces the dimensionality of the data while retaining most of the variance of the data. New variables are obtained, called principal components (which are a linear combination of the original variables), whose number is equal to or less than the initial number of variables, which explain most of the variance of the data and are independent of each other^50^. In this study, the number of retained PCA variables was chosen to explain at least ≥ 80% of the variance.

Two main PCA approaches were performed, using the **prcomp** function of R (https://www.rdocumentation.org/packages), including the scaling and centering procedures to have unit variance and a shift to zero, respectively, before analysis. In the first assessment, the “*1.HFD*, sex and TGN-20 effects on T2* and DTI at separated time points**”**, a PCA for each time (t0, t1 and t2) was performed independently. In the second approach, the “*2. T*_2_* and DTI follow up TGN-20 administration to HFD or CTRL mice”, a multiple factor analysis (MFA) was calculated. MFA is an extension of PCA that is used to handle data where the same variables (or “data tables”) are measured on different time-sets of observations^60^. Briefly, the procedure is achieved in two steps. First, a PCA of each data-table (or time-observation) is performed. Secondly, all the data tables are concatenated and a generalized PCA is done. With this approach, it is possible to study the time regimes that contribute most to each component derived from the PCA and report the influence of the variables from each time. To do that, we generated the Gl_short_-basal, Gl_long_-Gl_short_ and Gl_long−_basal groups of data by subtracting the corresponding values of MD, AD, RD, FA and T_2_* between the different time-points. MFA was performed using the mfa function of the FactoMiner package (https://www.rdocumentation.org/packages/FactoMineR/versions/2.9/topics/MFA). Two additional MFA were performed, either in the region of the hippocampus alone, or using the three remaining regions. In the MFA analysis, those animals that did not show valid DTI images for all time points, could not be included, and the corresponding groups were (i) CTRL fed and “saline + glc” (5 males and 5 females), (ii) CTRL and “TGN-020 + glc” (7 males and 5 females), (iii) HFD and “saline + glc” (5 males and 5 females), and (iv) HFD and “TGN-020 + glc” (3 males and 3 females).

In all cases, once the relevant PCA or MFA and predictors were identified, and to better understand the biological basis of the findings, we further investigated how the PCA-composing MRI variables were related to such predictors, by graphical representations and exploratory statistical tests (corrected for multiple comparisons).

### Statistical tests

Differences in BW were assessed by anova tests, with diet, sex and treatment as main effects, and their corresponding interactions, using the Anova function of the car package in R. The effects of diet, type of administration (“treatment”), brain region and sex, on the relevant PCA variables was perfomed by linear mixed-effect (lme) fitting^49^, using the lme function of the nlme package^61^, and mouse as a random intercept. An autoregressive correlation between regions was established (AR1 function of the nlme), in which the regions that are closer present more correlation. These lme models included fixed effects such as *region*, *diet*, *sex* and *treatment* and mouse as a random term. Interactions between different variables were added to explore possible joint effects, including double interactions (*Diet: Region*,* Diet: Sex*,* Treatment: Region*,* Treatment: Diet*,* Treatment: Sex* and *Sex: Region*), triple interactions (*Diet: Region: Sex*,* Diet: Region: Treatment*,* Region: Sex: Treatment* and *Sex: Treatment: Diet*) and the quadruple interaction (*Sex: Treatment: Diet: Region*). Next, Anova tests were performed to assess the statistical significance of the main effects and interactions on each PCA. For significant interactions, post-hoc contrasts were performed using the **emmeans** function (https://github.com/rvlenth/emmeans), and corresponding p-values adjusted for multiple comparisons with the Bonferroni method.

## Data Availability

The data presented in this study are available on request from the corresponding author due to the need for a formal data sharing agreement.
